# Supplementary material for: Learning curve and surgical outcome of robotic assisted colorectal surgery with ERAS program
Source: Sci Rep. 2022 Nov 29;12:20566. doi: 10.1038/s41598-022-24665-w (PMC9709162; doi:10.1038/s41598-022-24665-w)
Supplement: Supplementary file 1 — Supplementary Information 1. [file 41598_2022_24665_MOESM1_ESM.zip › LSC Raw data-Ñ[▒K/141/H_ñw╝╨░O▒Kñσ.pdf]

正式摘要(電子簽章：000000228045816)

W83 / W83 ☐ 英文標題(注意:無英文姓名-請補病人基本檔!) ☐【讀取】憑證

臺中榮民總醫院

出院病歷摘要

轉入號： 轉出號：

[redacted]

(8)流水編號	
(9)入院日期	110/10/11 (2021)
(10)轉科日期	
(11)出院日期	110/10/21 (2021) 住院天數計 10 日

診斷

- 入院 :Rectal Adenocarcinoma, moderately differentiated,cT1N1M0
  -s/p per-anal excision , pT1NxMx, arising in a tubular adenoma, submucosa(+)
  [Hx]
  1. ADHF, EF:25%(2020/10), favored coronary artery disease induced ischemic heart disease
  - LVEF:42% on 2021/10/12
  2. Coronary artery disease, MDCT showed LAD-M: total occlusion.
  3. Multiple GU/DU and colon-polyps under PPI
  4. fissure and hemorhoid 12" s/p hemorredectomy on 2021/05/05
- 出院 :Rectal Adenocarcinoma, moderately differentiated,cT1N1M0
  -s/p per-anal excision , pT1NxMx, arising in a tubular adenoma, submucosa(+)
  -s/p LAR with loop ileostomy on 2021/10/13
  pT1N0(0/11), MD, AI-, PI-, CRM-, disMg 1.5cm.
  [Hx]
  1. ADHF, EF:25%(2020/10), favored coronary artery disease induced ischemic heart disease
  - LVEF:42% on 2021/10/12
  2. Coronary artery disease, MDCT showed LAD-M: total occlusion.
  3. Multiple GU/DU and colon-polyps under PPI
  4. fissure and hemorhoid 12" s/p hemorredectomy on 2021/05/05

主訴

- Accidentally found rectal polyp during the colonscopy.

病史

- This is a 72-year-old male patient with history of
  1.Rectal cancer,cT1N1M0 s/p per-anal excision of tumor, patho: Adenocarcinoma, moderately differentiated, arising in a tubular adenoma. Tumor invades submucosa. Resection margins not involved, deep margin: -, 2mm, pT1NxMx.
  2. fissure and hemorrhoid 12" s/p hemorredectomy on 2021/05/05
  3. ADHF, EF:25%(2020/10), favored coronary artery disease induced ischemic heart disease
  - w/ Rt. pleural effusion, /p tapping
  4. Coronary artery disease, MDCT showed LAD-M: total occlusion.
  5. Multiple GU/DU and colon-polyps

  According to patient, he accidentally found rectal polyp during the colonscopy. Therefore, colonscopy EMR and per-anal excision was done on 2021/05. Pathology showed Adenocarcinoma, moderately differentiated, arising in a tubular adenoma. Tumor invades submucosa. Resection margins not involved, deep margin: -, 2mm, pT1NxMx. After discussing the pros and cons of laparoscopic low anterior resection, he decided to receive the operation for radical excision. We would arrange pre-op survey including lung function and 2D cardiac sonography.

  With the impression of Rectal cancer pT1NxMx, he was admitted for radical excision via laparoscopic low anterior resection.

體檢發現

-
  - General appearance :
    - easy looking , conscious clear , without cardiopulmonary distress
    - Vital signs自動帶入date 體溫(BT) 脈搏(P) 呼吸(R) 血壓(BP) 身高(BH) 體重(BW) BMI
      20211011 35.9C 66/min 18/min 149/68mmHg 155cm 55.8kg 23.2
    - Vital signs: B.P. 149/68 mmHg , P.R. 66 /min , R.R. 18 /min , B.T. 35.9 ℃
  - HEENT :
    - Eyes: no anemic conjunctiva, no icteric sclera, normal EOM
    - ENT: no otorrhea, no rhinorrhea, no erythematous mucosa, no injected tonsil, no deformity
  - Neck: supple, freely movable, trachea midline,no deformity, no engorged jugular vein , no palpable thyroid or lymph node enlargement
  - Chest:
    - Inspection: Grossly normal, symmetric and fully expansion, no accessory muscle used, no operation scar, no spider angiomata, no gynecomastia
    - Palpation: no palpable mass or nodule, no subcutaneous emphysema, no tenderness
    - Ausculation: clear breathing sound
  - Heart:
    - Palpation: no thrill, no heave
    - Percussion: normal heart size
    - Ausculation: regular heart beat, normal S1, S2, no gallop, no murmur
  - Extremities: No deformity, full ranged of motion without limitation, no clubbing finger or nail cyanosis, no pitting edema, no ulcer or pigmentation, no obviou

  CRS CONDITION

  - Karnofsky performance status: (100) Normal, no complaints, no evidence of disease
  - PE of Abdomen:
  - CEA: 3.49 ng/ml
  - Imaging Studies:
  - Colonoscope report: pending for 10/12
  - Sono , CT of abdomen and chest Films:檢驗項目 :CT, LUNG 報告日期 :1100927
    主 訴 :
    Result:Y Finding: CT of chest with IV contrast enhancement. > centrilobular emphysema over bilateral upper lung > A tiny subpleural GGN in LLL(3mm axi#58) > A small subpleural lung cyst over LLL (axi#52) > A 0.6 cm nodule in right lobe of thyroid, suggest follow up with ultrasound > A small cyst in S5 of liver. > Tortuosity and atherosclerotic changes of T-aorta. > calcifications at coronary arteries > bilateral renal cyst > No significant enlarged LNs over mediastinum. > No significant bony lesion over spine, ribs and sternum. > No enlargement of bil. adrenal glands. > Otherwise, no significant abnormal findings Impression: No obvious lung metastasis is noted.
    檢驗項目 :MRI - PELVIS 報告日期 :1100823
    主 訴 :
    Result:Y Finding: MRI of pelvis was performed and the images revealed: > History of rectal cancer S/P OP > No definite local tumor recurrence. Please correlate with clinical condition. > The previously mentioned metastatic lymphadenopathy at left perirectal space on prior 20210426 CT shows relatively stable in size, about 12 mm. Please correlate with clinical condition. > Some other small lymph nodes over pelvic and bilateral inguinal areas. Suggest clinical correlation and follow-up. > enlargement of prostate gland, R/O benign prostatic hyperplasia. Please correlate with clinical condition. > focal high signal intensity on T2WI with mild enhancement over right sacral area, R/O benign condition first, until proven otherwise. Impression: Please see descriptions.
  - Figures:
    1. colon & rectum

手術日期及方法

-

- 手術日期及方法20211013 1. Laparoscopic low anterior resection
  2. Intraoperative colonoscopy : (-)
  3. High ligation (+) : At IMA root. IMV and left colic artery were also ligated.
  4. EndoGIA (60-4.1 ; Green) X4 + EEA (29 mm) were used
  5. Proximal cut end: >10cm, distal cut end: 0.5cm
  6. One 15 Fr. Hemovac drainge tube was placed from LLQ of abdomen and inserted into the abdominal cavity
  7. The colon was not taken down from the splenic flexure site
  7. both circular donut intack, air leak test negative
  8. loop ileostomy was done over RLQ

住院治療經過

- After admission, pre-operation survey showed no contraindication for the operation. Thus, the operation(LAR with loop ileostomy) was performed smoothly on 2021/10/13. Hematuria was noted during the operation. His hematuria relieved gradually by hydration with transamin. He had massive amount of ostomy discharge(around 3000ml per day). Thus, we prescribed Loperamide1# Q6H and Dobecon 1# TID and his symptoms improved after that. We gradually titrate her diet from water to current warm III diet. Surgical pathology showed all negative for malignancy. There was no obstructive nor leakage sign. Due to relatively stable status, he may be discharged with OPD f/u.

合併症

一般檢查

- 累積報告 :

DATE	TIME	ALB	TP	BIL.T	BIL.D	ALKP	AST	ALT	LDH	AMY	LIPASE	NH3	Ferritin	UA
1101013	1520			0.5	0.1		16	11						
1101018	0905	3.3					12	5						

DATE	TIME	WBC	RBC	HGB	HCT	MCV	PLT	BLST	NEUT	BAND	SEG	LYM	MON	EOS	BAS
1101013	1522	15410	5.26	15.3	47.3	89.9	272		93.0			3.9	2.9	0.1	0.1
1101018	0903	10110	5.46	15.6	48.4	88.6	301		84.0			9.2	5.1	1.5	0.2

DATE	TIME	NA	K	CL	BUN	CREAT	eGFR	CA	Mg	P	CRP	ESR	PCT	D-Dimer	LACTATE
1101013	1520	141	3.4		9	0.81	99.56								
1101018	0905	137	3.8	102	10	0.92	85.95	7.9							

- 放射線報告 :檢驗項目 **:CHEST PA VIEW** 報告日期 **:1101013**

  主　訴：
  Result:N
  Finding:
  > Tortous thoracic aorta with Calcification over aortic knob.
  > The heart size is within normal limit.
  > increase in interstitial infiltrations at right lower lung fields
  > DJD changes with marginal spur formation and scoliosis of T-L spine.
  Impression:
  As above description.
  History Study Date: 20210331
  As above description.

  檢驗項目 **:CT, LUNG** 報告日期 **:1100927**

  主　訴：
  Result:Y
  Finding:
  CT of chest with IV contrast enhancement.
  > centrilobular emphysema over bilateral upper lung
  > A tiny subpleural GGN in LLL(3mm axi#58)
  > A small subpleural lung cyst over LLL (axi#52)
  > A 0.6 cm nodule in right lobe of thyroid, suggest follow up with ultrasound
  > A small cyst in S5 of liver.
  > Tortuosity and atherosclerotic changes of T-aorta.
  > calcifications at coronary arteries
  > bilateral renal cyst
  > No significant enlarged LNs over mediastinum.
  > No significant bony lesion over spine, ribs and sternum.
  > No enlargement of bil. adrenal glands.
  > Otherwise, no significant abnormal findings
  Impression:
  No obvious lung metastasis is noted.

  檢驗項目 **:MRI - PELVIS** 報告日期 **:1100823**

  主　訴：
  Result:Y
  Finding:
  MRI of pelvis was performed and the images revealed:
  > History of rectal cancer S/P OP
  > No definite local tumor recurrence. Please correlate with clinical condition.
  > The previously mentioned metastatic lymphadenopathy at left perirectal space on prior 20210426 CT shows relatively stable in size, about 12 mm. Please correlate with clinical condition.
  > Some other small lymph nodes over pelvic and bilateral inguinal areas. Suggest clinical correlation and follow-up.
  > enlargement of prostate gland, R/O benign prostatic hyperplasia. Please correlate with clinical condition.
  > focal high signal intensity on T2WI with mild enhancement over right sacral area, R/O benign condition first, until proven otherwise.
  Impression:
  Please see descriptions.

特殊檢查

病理報告

- 活體組織切片病理報告　手術日期：**110/10/13**　病理號：**S1032639**

  診斷醫師:文芙卿 報告日期:110/10/19
  Pathologic diagnosis:
  1. Rectum, low anterior resection --- Status post excision with scar and foreign body type giant cell reaction in lamina propria. No residual tumor seen. The bilateral cut ends and perirectal lymph nodes (0/11) are free of tumor.
  2. IMA root, dissection --- Unremarkable fibroadipose tissue and blood vessels. Negative for malignancy.
  3. IMV root, dissection --- Unremarkable fibroadipose tissue and blood vessels. Negative for malignancy.
  4. Rectum, cut end, distal, resection --- Negative for malignancy.
  Ancillary study for diagnosis:
  Step cut x 3 and CDX2 IHC stain done for section D, and target cells lost in IHC section.
  Prognostic and predictive factor:
  (Combined with previous excision specimen: S1015258)
  1. Tumor size: 0.8 cm. (in S1015258)
  2. Depth of invasion: Submucosa. (in S1015258)
  3. Histologic grade: G2: Moderately differentiated. (in S1015258)
  4. Angiolymphatic invasion: Not identified.
  5. Perineural invasion: Not identified.
  6. Tumor deposits (discontinuous extramural extension): Not identified.
  7. Macroscopic tumor perforation: Not identified.
  8. Surgical Margins:

- a) Proximal Margin: Uninvolved by invasive carcinoma.
  b) Distal Margin: Uninvolved by invasive carcinoma.
  c) Radial or Mesenteric Margin: Uninvolved by invasive carcinoma.
  9. Lymph node status: Uninvolved (0/11).
  10. Extranodal involvement: Not identified.
  11. Tumor regression grading S/P CCRT: No known presurgical therapy.
  12. Type of polyp in which invasive carcinoma arose: Not identified.
  13. Additional pathologic findings: Not identified.
  14. Pathological TNM Stage: pT1N0 (According to the eighth edition, American Joint Committee on Cancer Staging Guidelines for Tumors).
  15. TNM descriptors: Not applicable.
  Gross description:
  The specimen consists of 1) a segment of colon, 16 cm in length and 6.5 cm in proximal cut end circumference, 8 cm in distal circumference, and perirectal soft tissue. A surgical stitch marked the previous tumor excision site, which is 1.5 cm from distal cut end and 13.5 cm from proximal cut end. On cross section, there is no mass lesion found grossly. There are two stitches noted on perirectal soft tissue margin, which labeled as "MRI LN+". On cut, there is no enlarged lymph node found in adjacent soft tissue. The both cut ends are unremarkable grossly. 2) IMA root, a piece of tan yellow soft tissue, 2.5x1.6x1.5 cm. 3) a piece of tan soft tissue, 2.6x1.3x1 cm, labeled as "IMV root". 4) distal cut end, a piece of tan mucosa-covered soft tissue, 2x1.8x1 cm. Representative part for section: A) proximal cut end B) distal cut end C-D) colon near one stitch E) radial margin and soft tissue near two stitches F) all of specimen 2 G-H) specimen 3-4. Blue ink indicated serosa; yellow ink indicated radial margin.
  #T-59600_2 #M-78060_2 0 1105 SP0000
  #T-C4560_2 #M-09183_2 0 1105 000000

  活體組織切片病理報告　手術日期：**109/10/12**　病理號：**S0931901**

  診斷醫師:陳志榮 報告日期:109/10/13
  Pathologic diagnosis:
  Stomach, antrum, endoscopic biopsy --- Chronic active gastritis with intestinal metaplasia.
  Ancillary study for diagnosis:
  Giemsa stain for Helicobacter Pylori identification done.
  Prognostic and predictive factor:
  Helicobacter-like microorganisms (+) found.
  Gross description:
  The specimen consists of 3 pieces of tan soft tissue, up to 0.5x0.2x0.2 cm. All for section.
  Microscopic description:
  1. Neutrophils activity- Moderate.
  2. Chronic inflammation/Mononuclear cells infiltration- Marked.
  3. Atrophy of glands- Moderate.
  4. Intestinal metaplasia- Moderate.
  #T-57000_2 #M-42000_2 0 1340 00HP00
  #T-57000_2 #M-73320_2 0 1340 000000

其它

出院時情況

- 3.改門診治療

出院指示

藥名	劑量	劑量單位	用法	頻次	數量	數量單位	現況	天數	單位
Anxiedin tab 0.5mg	1	TAB	PO	QNPRN	5	TAB	TH	5	天
Loperamide cap 2mg	1	CAP	PO	QD	5	CAP	TH	5	天
Dobecon tab 100mg	1	TAB	PO	TIDAC	15	TAB	TH	5	天
OCAS Harnalidge 0.4 mg	1	TAB	PO	QDAC	5	TAB	TH	5	天
Esomeprazole tab	1	TAB	PO	QDAC	28	TAB	TH	28	天
Ferrous Gluco-B	1	TAB	PO	TIDAC	84	TAB	TH	28	天
IsormoL tab 20mg	1	TAB	PO	BID	56	TAB	TH	28	天
(□)Rosis tab 40mg	1	TAB	PO	QD	28	TAB	TH	28	天
100mg Entresto tab	1	TAB	PO	BIDPC	56	TAB	TH	28	天
Spironolactone-25	1	TAB	PO	QD	28	TAB	TH	28	天
Ivabradine tab 5mg	0.5	TAB	PO	BIDCC	28	TAB	TH	28	天
5mg Bisoprolol tab	0.5	TAB	PO	QD	14	TAB	TH	28	天

出院計劃

- 門診日期
  - date on 2021/10/25

主治醫師 2222H 林俊余 / 住院醫師 9727A 林順鴻

正式摘要(電子簽章：**000000228045816)**

W83 / W83

doctorRank:B
